# Oxygenated VOC Detection Using SnO_2_ Nanoparticles with Uniformly Dispersed Bi_2_O_3_

**DOI:** 10.3390/nano14242032

**Published:** 2024-12-18

**Authors:** Haoyue Yang, Koichi Suematsu, Felipe Hiroshi Mashiba, Ken Watanabe, Kengo Shimanoe

**Affiliations:** 1 Interdisciplinary Graduate School of Engineering Sciences, Kyushu University, Kasuga 816-8580, Fukuoka, Japan; yang.haoyue.788@s.kyushu-u.ac.jp (H.Y.); mashiba.felipe.hiroshi.080@s.kyushu-u.ac.jp (F.H.M.); 2 Department of Advanced Materials Science and Engineering, Faculty of Engineering Sciences, Kyushu University, Kasuga 816-8580, Fukuoka, Japan; watanabe.ken.331@m.kyushu-u.ac.jp

**Keywords:** SnO_2_ nanoparticles, Bi_2_O_3_-loading, oxygenated VOC detection, interface, surface oxygen ions

## Abstract

Bi_2_O_3_ particles are introduced as foreign additives onto SnO_2_ nanoparticles (NPs) surfaces for the efficient detection of oxygenated volatile organic compounds (VOCs). Bi_2_O_3_-loaded SnO_2_ materials are prepared via the impregnation method followed by calcination treatment. The abundant Bi_2_O_3_/SnO_2_ interfaces are constructed by the uniform dispersion of Bi_2_O_3_ particles on the SnO_2_ surface. The results of oxygen temperature-programmed desorption suggest that Bi_2_O_3_-loaded SnO_2_ samples display improved surface oxygen ions than neat-SnO_2_ NPs. As a result, the gas sensor based on 1 mol% Bi_2_O_3_-loaded SnO_2_ (1Bi-L-SnO_2_) composites shows significantly higher sensitivity and a faster response speed toward various oxygenated VOCs compared with SnO_2_, especially at 200 °C and 250 °C. The results of catalytic combustion and temperature-programmed reaction measurements reveal the dominant role of adsorption and partial oxidation during ethanol combustion on SnO_2_ and 1Bi-L-SnO_2_ surfaces. In this case, the improvement in the sensing performance of the 1Bi-L-SnO_2_ sensor can be associated with the increase in surface oxygen ions at Bi_2_O_3_/SnO_2_ interfaces. The results confirm the significant role of surface functionalization for sensing materials. The obtained outstanding sensing performance provides the potential application for the simultaneous detection of total oxygenated VOCs in practice.

## 1. Introduction

As an important group of volatile organic compounds (VOCs), oxygenated VOCs (including organic alcohols, aldehydes, and ketones, etc.) are widespread in the atmosphere derived from various human activities and biogenic emissions, such as the oxidation of hydrocarbons, emission of oxygenated fuels in industrial application, burning of biomass, etc. [1,2,3]. The various oxygenated VOCs lead to the formation of ozone and secondary organic aerosols, resulting in the threat to environmental pollution and human health [4,5]. In addition, many kinds of oxygenated VOCs are concomitant and interconvertible during the chemical reactions [6,7]. Hence, the importance and necessity of achieving simultaneous detection of total oxygenated VOCs in the atmosphere is noticeable. Chemiresistive-type gas sensors using semiconductor metal oxides (SMOs) like SnO_2_, ZnO, WO_3_, and In_2_O_3_ have garnered many outstanding performances for various gas detection in various applications including air quality monitoring, industrial safety, and so on [8,9,10,11,12]. In particular, SnO_2_ has been considered as one of the most sensitive materials by continuous research for decades [13,14,15]. At present, researchers have proposed various types of SnO_2_-based gas sensors for the efficient detection of specific oxygenated VOCs, such as ethanol, acetone, formaldehyde, and so on [16,17,18]. However, it is still insufficient for the detection of total oxygenated VOCs in the practical application. This highlights the importance of exploring sensing material to detect total oxygenated VOCs.

The basic understanding of the sensing mechanism is crucial for the research of gas sensors. It is normally recognized that the sensitivities of SnO_2_-based resistive-type gas sensors to fundamental gases such as CO and H_2_ are evaluated by the change in electrical resistances arising from the combustion reaction between gas molecules and adsorbed oxygen ions to form CO_2_ and H_2_O on the surface of materials. However, many studies indicate the complete combustion of oxygenated VOCs on the surfaces of metal oxides tends to occur under high operating temperatures (almost above 300 °C), and considerable intermediates will be produced during the combustion reaction [19,20,21]. Interestingly, the reported research has indicated the considerable sensitivity of SnO_2_-based gas sensors to ethanol at 250 °C [22]. In this case, the detection mechanism for oxygenated VOCs at temperatures below 300 °C may be different from high temperatures. Therefore, it is necessary to investigate the basic reaction process during oxygenated VOC combustion on SnO_2_-based gas sensors at various temperatures.

According to reported research, the sensing performance of SnO_2_-based gas sensors can be further improved after surface functionalization by introducing foreign additives, such as metal oxides and noble metals [9,23,24,25,26]. Generally, the functionalized surface properties of SnO_2_ by foreign additives mainly include the modulation of active oxygen ions, acidic sites, etc. The former parameter is directly responsible for the activity of the oxidation reaction of target gas molecules. The surface acid–base property concerns the specific adsorption and selective conversion of target gas molecules on the material surface [27,28,29]. Thus, the sensing properties of SnO_2_-based gas sensors to oxygenated VOCs may be further improved by modulating the surface properties using foreign additives. α-Bi_2_O_3_ particles have stood out for their high stability and environmental promise, conducing to the application as a photocatalyst, selective oxidation catalyst, sensing material, etc. [30,31,32]. In particular, the Bi atoms in Bi_2_O_3_ contribute to the electrical conductivity due to the hybridization between the O 2p orbital and Bi 6s or 6p orbitals [33]. Several studies have confirmed the facilitation of the sensing performance of SMOs-based gas sensors by employing Bi_2_O_3_ particles as foreign additives [34,35,36]. Moreover, it is possible that Bi_2_O_3_ shows different acid–base properties with SnO_2_ due to the different electronegativity of Bi^3+^ (13.3) than Sn^4+^ (16.2) [37]. Consequently, Bi_2_O_3_ may be a promising candidate as a foreign additive to improve the sensing properties of SnO_2_-based gas sensors to oxygenated VOCs.

Herein, in order to improve the selectivity to typical oxygenated VOCs, we employed Bi_2_O_3_ particles as foreign additives on a SnO_2_-based gas sensor. Bi_2_O_3_-loaded SnO_2_ materials were synthesized, and Bi_2_O_3_ was uniformly dispersed on the surface of SnO_2_ nanoparticles (NPs). Bi_2_O_3_-loaded SnO_2_ materials showed increased surface oxygen ions than SnO_2_ NPs. As a result, the gas sensor based on 1 mol% Bi_2_O_3_-loaded SnO_2_ nanoparticles showed excellent sensitivities toward various oxygenated VOCs, especially at 200 °C and 250 °C. Meanwhile, catalytic combustion and temperature-programmed reaction measurements revealed that the adsorption–desorption, dissociation, and partial oxidation of oxygenated VOCs were dominant at temperatures lower than 300 °C. In conclusion, the improvement in sensing properties of the SnO_2_ sensor by Bi_2_O_3_-loading could be ascribed to the increased surface oxygen ions at the contact interfaces between Bi_2_O_3_ and SnO_2_ accelerated the adsorption and partial combustion of oxygenated VOCs. This research provided the basic insight into the reaction mechanism of oxygenated VOCs at low temperatures (below 300 °C). Meanwhile, the results indicated the uniform dispersion of foreign additives played an active role in the sensing properties of gas sensors.

## 2. Materials and Methods

### 2.1. Materials Synthesis

**The synthesis of pure SnO_2_ NPs.** SnO_2_ NPs were synthesized via hydrothermal synthesis accompanied by calcination. Firstly, 1 M of SnCl_4_·5H_2_O (98.0%, special grade; FUJIFILM Wako Pure Chemical Corporation, Osaka, Japan) solution was dropwise added into 1 M of NH_4_HCO_3_ (99.0%, special grade; FUJIFILM Wako Pure Chemical Corporation, Osaka, Japan) solution under stirring. Next, the obtained stannic acid gel was washed to remove Cl^−^ and then mixed with deionized water. After adjusting the PH to 10.5 by tetramethylammonium hydroxide (15%, special grade, FUJIFILM Wako Pure Chemical Corporation, Osaka, Japan) solution, the mixture was heated in a 100 mL stainless steel reactor with Teflon inner cylinder at 200 °C for 10 h in an oven. Subsequently, the obtained transparent sol was dried at 120 °C and annealed at 600 °C for 3 h under O_2_ flow to prepare SnO_2_ NPs.

**The synthesis of Bi_2_O_3_-mixed SnO_2_ samples.** Firstly, Bi_2_O_3_ particles were obtained by calcining the Bi(NO_3_)_3_·5H_2_O (99.5 %, special grade, KISHIDA Chemical Co. Ltd., Osaka, Japan) at 550 °C for 3 h under O_2_ flow. Next, 1.5 g SnO_2_ NPs and a stoichiometric amount of Bi_2_O_3_ powders were mixed and ground manually for 15 min. Then the particles were calcined at 530 °C for 3 h under O_2_ flow to obtain Bi_2_O_3_-mixed SnO_2_ materials, which were labeled as 1Bi-M-SnO_2_ and 3Bi-M-SnO_2_ according to the concentration of Bi_2_O_3_ (the atomic ratio of Bi to Sn was 1/100 and 3/100, respectively).

**The synthesis of Bi_2_O_3_-loaded SnO_2_ samples.** Bi_2_O_3_-loaded SnO_2_ samples with various Bi contents were synthesized by a simple impregnation method. 1.5 g SnO_2_ NPs dissolved in 20 mL deionized water was impregnated on an aqueous solution of a stoichiometric amount of Bi(NO_3_)_3_·5H_2_O with stirring for 24 h at room temperature. The resulting precipitates were washed by centrifugation, dried at 100 °C, and calcined at 550 °C for 3 h under O_2_ flow. The obtained samples were referred to hereafter as 1Bi-L-SnO_2_ and 3Bi-L-SnO_2_ (the atomic ratio of Bi to Sn was 1/100 and 3/100, respectively).

### 2.2. Material Characterization

Wavelength-dispersive X-ray fluorescence spectroscopy (WDX, Supermini 200, Rigaku, Tokyo, Japan) was used to evaluate the content of Bi ions in Bi_2_O_3_-loaded SnO_2_ materials. The crystal structures of as-synthesized SnO_2_, Bi_2_O_3_-mixed SnO_2_, and Bi_2_O_3_-loaded SnO_2_ samples were investigated by X-ray diffractometry (XRD; MiniFlex, Rigaku, Tokyo, Japan) with CuKα radiation. The specific surface reaction and pore volume of materials were respectively evaluated by N_2_ adsorption/desorption analyzer (BELSORP-mini II, MicrotracBEL Corp., Osaka, Japan), and calculated by Brunauer–Emmett–Teller (BET) method and Barrett–Joyner–Halenda (BJH) method, respectively. The distribution of Bi_2_O_3_ on the surface of SnO_2_ was investigated by scanning electron microscopy (SEM; JCM-7000, JEOL, Tokyo, Japan) equipped with an energy-dispersive X-ray spectroscopy (EDS) attachment.

Temperature-programmed desorption of oxygen and ammonia (O_2_-TPD and NH_3_-TPD) measurements were expected to estimate the adsorption–desorption of oxygen and NH_3_ on sample surfaces using a catalyst analyzer (BELCAT II, MicrotracBEL Corp., Osaka, Japan) equipped with a thermal conductivity (TCD) detector. The system is also linked with a quadrupole mass spectrometer (QMS, BELMASS II, MicrotracBEL Corp., Osaka, Japan) to analyze the desorbed products emitted from the material surface. The gas adsorption–desorption on the materials’ surfaces was analyzed using temperature-programmed reaction (TPR) measurement. The TPR system consisted of a gas mixing system, a reaction chamber, and a gas detector with a quadrupole mass spectrometer (QMS; PrismaPlus QMG220, PFEIFFER, Hessen, Germany). The catalytic combustion measurement was employed to monitor the consumption of gas molecules and production of intermediates caused by gas combustion on material surfaces using a self-assembled system consisting of a gas mixing system, reaction chamber, and gas chromatography (GC-4000 Plus, GL Science Inc., Tokyo, Japan) equipped with a flame ionization detector and a methanizer (MT221), followed by the connection with series to 2 m of Porapak Q and Porapak N columns (GL Science Inc., Tokyo, Japan). The details of measuring processes were described in Supporting Information.

### 2.3. Sensor Fabrication and Measurement

The gas sensors were fabricated by screen printing method. Firstly, Au electrodes were screen-printed on an alumina substrate (9 × 13 × 0.38 mm^3^) followed by heat-treated at 850 °C for 3 h (line width: 180 μm, distance between lines: 90 μm, sensing area: 64 mm^2^). Next, the samples were mixed with α-terpineol to form paste, and then screen-printed on the alumina substrate with Au electrodes. The resulting devices were calcined at 500 °C for 3 h in the air to remove the organic binder. The obtained gas sensors were placed in an electric furnace combined with a gas flow apparatus under the total flow rate of 100 cm^3^/min controlled by mass flow controllers. The operating temperatures of gas sensors were modulated at 200 °C, 250 °C, 300 °C, and 350 °C. Each sensor was connected in series to a standard resistor and applied a DC voltage of 4 V. An electrometer (2701; Keithley Instruments, Solon, OH, USA) was employed to measure the electrical resistances by evaluating the voltage across the standard resistor. The sensor response was evaluated by the change in electrical resistances in synthesis air and target gas atmospheres (*S* = *R*_a_/*R*_g_). The response time was considered as the time required for the sensor to be 90% of maximum response change [38]. The target gases were diluted by N_2_ with 21% O_2_ to obtain the tested concentration (5 ppm) under a gas mixture system.

## 3. Results and Discussion

### 3.1. Materials Characteristics

**Figure 1:**
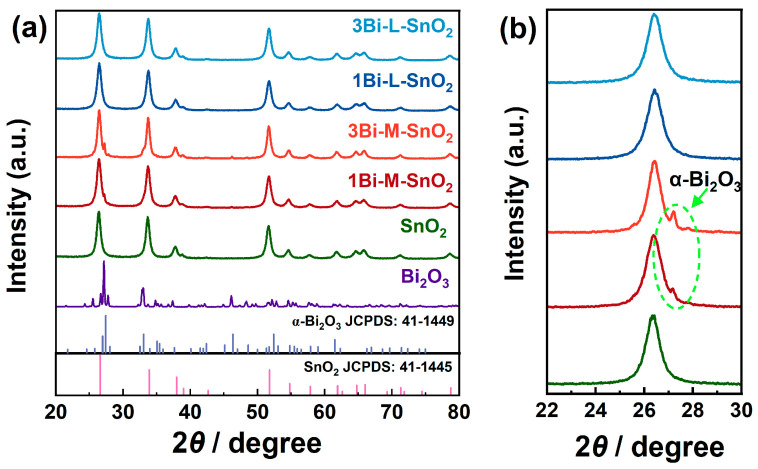
(**a**) XRD patterns of SnO_2_, 1Bi-M-SnO_2_, 3Bi-M-SnO_2_, 1Bi-L-SnO_2_, 3Bi-L-SnO_2_; (**b**) the corresponding magnified region at 22–30°.

The actual concentration of Bi ions in Bi_2_O_3_-loaded SnO_2_ materials was evaluated by WDX analysis using the calibration results of Bi_2_O_3_-mixed SnO_2_ materials. The calculated amounts of Bi ions in 1Bi-L-SnO_2_ and 3Bi-L-SnO_2_ materials were 1.1 mol% and 2.8 mol%, respectively. The results indicated the comparable content of Bi ions in Bi_2_O_3_-loaded SnO_2_ and Bi_2_O_3_-mixed SnO_2_ materials. The XRD patterns of SnO_2_, Bi_2_O_3_-loaded SnO_2_, and Bi_2_O_3_-mixed SnO_2_ materials (Figure 1) showed diffraction peaks matched well with tetragonal rutile structure SnO_2_ with the space group of *P*4_2_/mnm (JCPDS: 41-1445), indicating all as-synthesized materials had the same SnO_2_ crystal phase. Meanwhile, the obtained Bi_2_O_3_ particles were assigned to monoclinic α-Bi_2_O_3_ with the space group of *P*2_1_/c (JCPDS: 41-1449). The diffraction peak appeared at about 27.15° of Bi_2_O_3_-mixed SnO_2_ samples could be assigned to the (111) plane of α-Bi_2_O_3_, proving that α-Bi_2_O_3_ particles were successfully complexed on the surface of SnO_2_. No diffraction peak of Bi_2_O_3_ was observed on Bi_2_O_3_-loaded SnO_2_ samples, which might be caused by the well-dispersion of Bi_2_O_3_ particles. In addition, the average crystallite sizes of SnO_2_ in as-prepared materials were calculated by the Scherrer formula, as shown in Table S1. The results indicated the nanostructure of obtained SnO_2_ powders. There was no obvious change in crystallite size after Bi_2_O_3_-mixing and Bi_2_O_3_-loading on SnO_2_ NPs.

**Figure 2:**
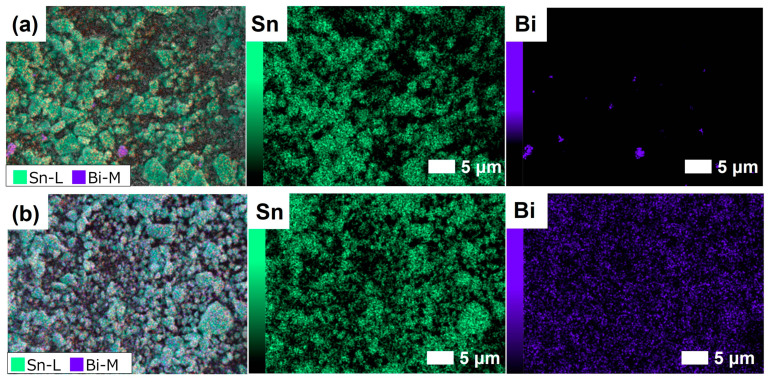
SEM-EDS elemental mapping images of Sn and Bi for (**a**) 1Bi-M-SnO_2_ and (**b**) 1Bi-L-SnO_2_ samples.

To investigate the distribution of Bi_2_O_3_ on the SnO_2_ surface, we detected the SEM-EDS elemental mapping images of Sn and Bi in Bi_2_O_3_-mixed and Bi_2_O_3_-loaded SnO_2_ materials, as shown in Figure 2 and Figure S2. For Bi_2_O_3_-mixed SnO_2_ materials (Figure 2a and Figure S2a), Sn was observed almost across the whole imaged area, while Bi was exhibited only in localized regions. Meanwhile, there was little overlapping area between Bi and Sn, proving the separate distribution of Bi_2_O_3_ and SnO_2_ particles with little contact interfaces in Bi_2_O_3_-mixed SnO_2_ materials. In contrast, Figure 2b and Figure S2b revealed the uniform distribution of Sn and Bi elements in Bi_2_O_3_-loaded SnO_2_ materials, indicating Bi_2_O_3_ particles were homogeneously dispersed on the surface of SnO_2_ NPs. As a result, abundant contact interfaces at Bi_2_O_3_/SnO_2_ were constructed in Bi_2_O_3_-loaded SnO_2_ materials, while major Bi_2_O_3_ particles were agglomerated and physically mixed with SnO_2_ NPs in Bi_2_O_3_-mixed SnO_2_ materials. The specific surface area and average pore volume of Bi_2_O_3_-mixed SnO_2_ materials were comparable to those of Bi_2_O_3_-loaded SnO_2_ and slightly larger than that of neat-SnO_2_ (Table S1). The indistinctive impact on the surface area and porosity of SnO_2_ NPs after introducing Bi_2_O_3_ particles might be associated with the low concentration of Bi_2_O_3_ particles, and the observed slight increase in neat-SnO_2_ might be ascribed to the difference during the sample preparation process.

### 3.2. Gas Sensing Properties

**Figure 3:**
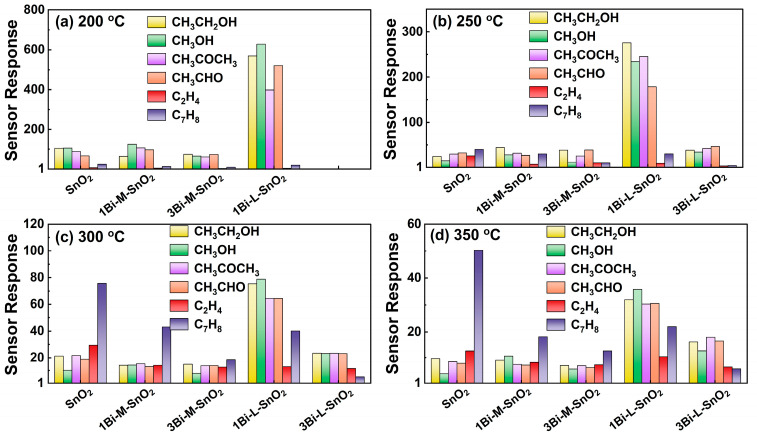
The responses of as-fabricated gas sensors to 5 ppm various VOCs at (**a**) 200 °C, (**b**) 250 °C, (**c**) 300 °C, and (**d**) 350 °C.

Figure S3 showed the electrical resistance in synthetic air (*R*_a_) of neat-SnO_2_, Bi_2_O_3_-mixed SnO_2_, and Bi_2_O_3_-loaded SnO_2_ gas sensors at 200 °C, 250 °C, 300 °C, and 350 °C. It was difficult to detect the sensing performance of the 3Bi-L-SnO_2_ sensor at 200 °C due to the excessively high electrical resistance exceeding system limits. The *R*_a_ of Bi_2_O_3_-loaded SnO_2_ sensors were larger than that of neat-SnO_2_ and Bi_2_O_3_-mixed SnO_2_ sensors at all detected temperatures, confirming the effect of uniform dispersion of Bi_2_O_3_ particles on the conductivity of SnO_2_-based sensor. We investigated the sensitivities of gas sensors to 5 ppm of various VOCs (CH_3_CH_2_OH, CH_3_OH, CH_3_COCH_3_, CH_3_CHO, C_2_H_4_, and C_7_H_8_) at 200–350 °C. As displayed in Figure 3, the gas sensors based on Bi_2_O_3_-mixed SnO_2_ samples showed similar responses to tested oxygenated VOCs (CH_3_CH_2_OH, CH_3_OH, CH_3_COCH_3_, CH_3_CHO) with neat-SnO_2_ sensor, indicating the agglomerated Bi_2_O_3_ particles exhibited little effect on the reactivity of SnO_2_ for oxygenated VOCs detection. Meanwhile, the 1Bi-L-SnO_2_ sensor showed dramatically improved sensitivities to tested oxygenated VOCs at all detected operating temperatures (especially at 200 °C and 250 °C). The phenomenon confirmed the uniform dispersion of Bi_2_O_3_ particles on the SnO_2_ surface during the Bi_2_O_3_-loading process played a vital role in the adsorption and combustion of oxygenated VOCs. However, the responses of the 3Bi-L-SnO_2_ sensor to oxygenated VOCs were lower than that of the 1Bi-L-SnO_2_ sensor, which might be attributed to the excessive dispersion of Bi_2_O_3_ particles prevented the diffusion of gas molecules into the sensing layer of SnO_2_ [39]. Besides, the neat-SnO_2_ sensor showed clearly higher responses to C_2_H_4_ and C_7_H_8_ than Bi_2_O_3_-loaded SnO_2_ and Bi_2_O_3_-mixed SnO_2_ sensors. In conclusion, the 1Bi-L-SnO_2_ sensor showed excellent sensitivity and selectivity to oxygenated VOCs, especially at 200 °C and 250 °C. Additionally, the responses toward oxygenated VOCs were decreased with the increase in temperature.

**Figure 4:**
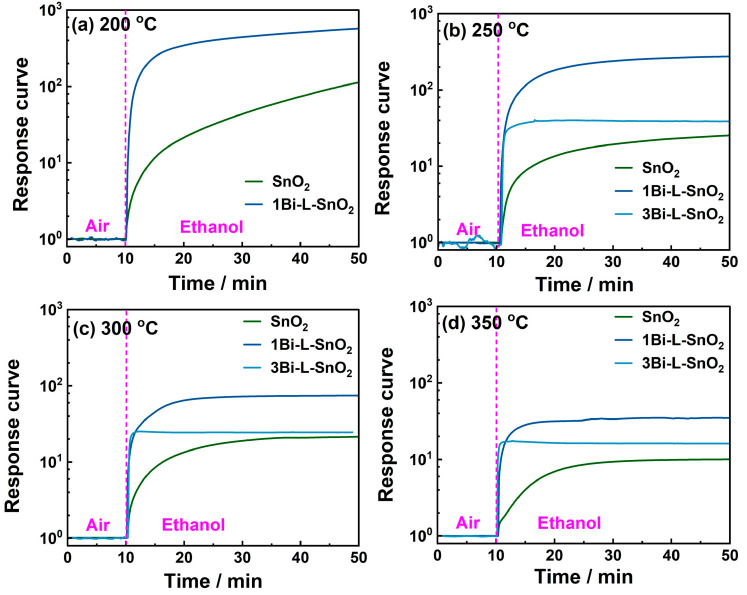
Dynamic time-dependence response curves of SnO_2_ and Bi_2_O_3_-loaded SnO_2_ sensors to 5 ppm of ethanol at (**a**) 200 °C, (**b**) 250 °C, (**c**) 300 °C, and (**d**) 350 °C.

The response curve is a crucial parameter to estimate the sensing performance of gas sensors. The dynamic time-dependence response curves of obtained gas sensors based on SnO_2_ and Bi_2_O_3_-loaded SnO_2_ materials toward ethanol as the representative of tested oxygenated VOCs at 200–350 °C are shown in Figure 4. The corresponding response time required for the sensors to 90% of response changes are calculated in Table S2. Figures S4–S7 exhibited the dynamic response curves of all the obtained gas sensors toward tested oxygenated VOCs at 200–350 °C. Clearly, the responses of all sensors were rapidly increased during the initial period under the target gases’ atmosphere. Generally, the initial rapid increase in response could be associated with the consumption of adsorbed oxygen ions on the surface of sensing materials. Bi_2_O_3_-loaded SnO_2_ sensors showed an obviously improved increasing tendency at the initial period compared to neat-SnO_2_ sensors. Moreover, the response time of SnO_2_ to ethanol was decreased with the rising Bi_2_O_3_-loading content. In this case, the improved response speed of Bi_2_O_3_-loaded SnO_2_ sensors to ethanol might be assigned to the improved amount of surface oxygen ions of Bi_2_O_3_-loaded SnO_2_ surfaces. The response speeds of all sensors were increased with the rising temperature, corresponding to the enhanced reactivity of surface oxygen ions of sensing materials. Consequently, the rapid response and outstanding sensitivity to oxygenated VOCs combined with weak responses to C_2_H_4_ and C_7_H_8_ of the 1Bi-L-SnO_2_ sensor proved the potential for sensitive and selective oxygenated VOCs detection. Additionally, the results indicated the different distribution of Bi_2_O_3_ particles on the SnO_2_ surface caused by Bi_2_O_3_-mixing and Bi_2_O_3_-loading processes showed various sensing performances, the abundant Bi_2_O_3_/SnO_2_ interfaces formed by the uniform dispersion of Bi_2_O_3_ particles might be the crucial parameter improving the sensing properties.

### 3.3. Catalytic Combustion Measurement

**Figure 5:**
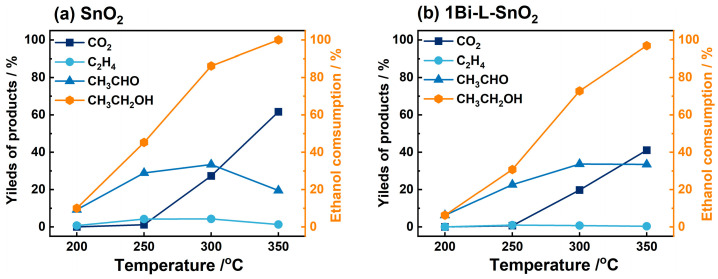
The temperature-dependence consumption of CH_3_CH_2_OH and the yields of products (CO_2_, CH_3_CHO, and C_2_H_4_) during ethanol combustion on the surfaces of (**a**) SnO_2_ and (**b**) 1Bi-L-SnO_2_ particles from 200 °C to 350 °C.

Generally, the combustion of gas molecules on material surfaces plays a vital role in the sensing performance of gas sensors. As one of the typical oxygenated VOCs, the combustion of ethanol on neat-SnO_2_ and 1Bi-L-SnO_2_ surfaces under 200–350 °C was investigated, as shown in Figure 5. It was observed that the consumption of ethanol gradually increased from 200 °C and approximately reached 100% at 350 °C. Simultaneously, a number of CH_3_CHO and a little of C_2_H_4_ were produced during 200–350 °C, which were caused by the dehydrogenation and dehydration of ethanol, respectively. Noteworthy, no CO_2_ was detected at 200 °C and 250 °C, indicating the absence of complete combustion of ethanol. Hence, ethanol molecules were initially adsorbed on material surfaces and then converted to CH_3_CHO combined with a little C_2_H_4_. Subsequently, CH_3_CHO molecules were oxidized to intermediates (CH_3_COOH, CO, etc.) [40,41]. With the further increase in temperature, CO_2_ was gradually produced by the complete combustion of ethanol. Meanwhile, the production of CO_2_ was about 27% and 61% at 300 °C and 350 °C, revealing the presence of both partial and complete combustion of ethanol on the SnO_2_ surface. Besides, the productions of CO_2_ and C_2_H_4_ on 1Bi-L-SnO_2_ were smaller than those on the SnO_2_ surface, as demonstrated in Figure S8. As reported previously, ethanol molecules could be converted to CH_3_CHO and C_2_H_4_ on basic and acid material surfaces, respectively [19]. Hence, the almost disappeared yield of C_2_H_4_ on the 1Bi-L-SnO_2_ surface might be ascribed to the reduced surface acidity. Furthermore, the decreased CO_2_ production corresponded to the declined complete combustion of ethanol. Consequently, the complete combustion of ethanol to CO_2_ on the SnO_2_ surface was weakened after Bi_2_O_3_-loading, and CH_3_CHO became the main intermediate combined with the reduced production of C_2_H_4_. As a result, partial combustion was dominant during the combustion of ethanol and CH_3_CHO on the surface of 1Bi-L-SnO_2_ material below 300 °C. In addition, the combustion of acetone on SnO_2_ and 1Bi-L-SnO_2_ surfaces was investigated in Figure S9. It was obvious that no acetone consumption and CO_2_ yields were detected at 200 °C and 250 °C, suggesting little combustion of acetone occurred on material surfaces. As the temperature increased to 300 °C and 350 °C, part of the acetone was combusted to CO_2_, a little CH_3_CHO, and other intermediates. Similarly, neat-SnO_2_ showed a higher amount of acetone consumption and CO_2_ production than 1Bi-L-SnO_2_, indicating the improvement in partial combustion of acetone after Bi_2_O_3_-loading. As a result, the combustion of acetone on the 1Bi-L-SnO_2_ surface exhibited a similar phenomenon to that of ethanol, which was mainly in accordance with partial combustion.

### 3.4. TPD and TPR Measurements

**Figure 6:**
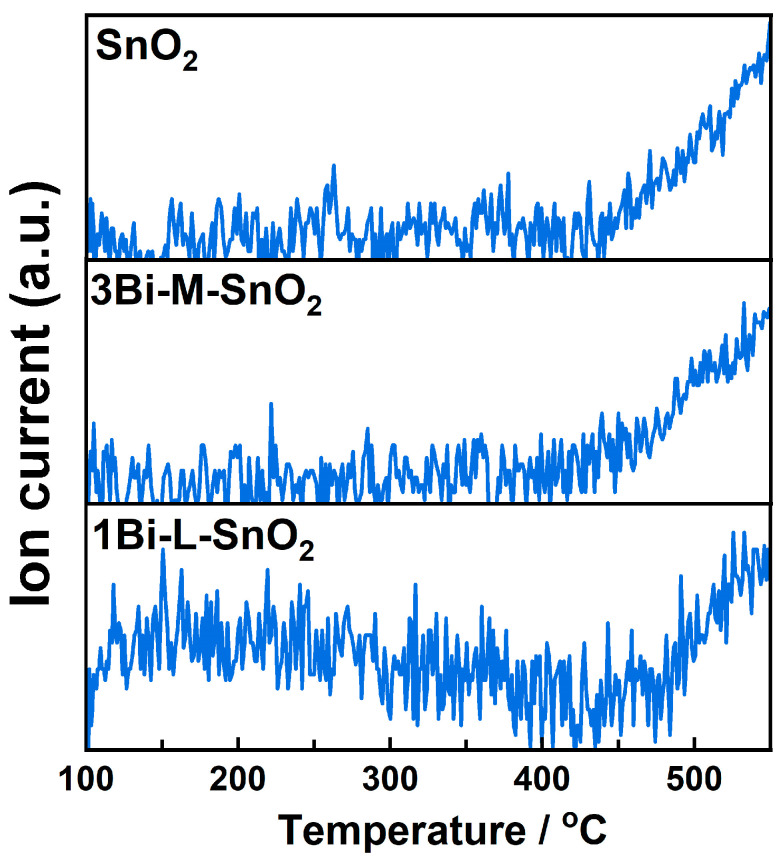
O_2_-TPD combined with mass spectrometer monitored at *m*/*z* 32 of SnO_2_, 3Bi-M-SnO_2_, and 1Bi-L-SnO_2_ materials.

The desorption of oxygen on the surfaces of SnO_2_, 3Bi-M-SnO_2_, and 1Bi-L-SnO_2_ materials was evaluated by O_2_-TPD combined with a mass spectrometer monitored at *m*/*z* 32, as depicted in Figure 6. Obviously, the desorption signal of SnO_2_ gradually increased from approximately 450 °C, corresponding to the desorption of surface lattice oxygen (O^2−^) of SnO_2_ [16,42]. 3Bi-M-SnO_2_ sample showed a similar phenomenon with neat-SnO_2_, indicating the agglomerated Bi_2_O_3_ showed little effect on the desorption of oxygen of SnO_2_. For 1Bi-L-SnO_2_, the temperature-dependent desorption signal could be roughly categorized as 100–400 °C and 400–550 °C. The former signal was assigned to the desorption of active oxygen ions (such as O_2_^−^, O^−^, and O^2−^) [43,44]. The desorption at higher temperatures was caused by the desorption of O_2_ arising from the surface lattice oxygen of SnO_2_. Consequently, the Bi_2_O_3_-loading onto the SnO_2_ surface facilitated the desorption of surface oxygen ions during 100–400 °C, which might be attributed to the abundant Bi_2_O_3_/SnO_2_ interfaces. Additionally, the effect of Bi_2_O_3_ particles on the surface acidity of SnO_2_ NPs was investigated by NH_3_-TPD measurements. As demonstrated in Figure S10, the desorption amount of NH_3_ on the SnO_2_ surface was decreased after Bi_2_O_3_-mixing and Bi_2_O_3_-loading processes, suggesting the exposure of acid sites on the surface of SnO_2_ was declined by the introduction of Bi_2_O_3_ particles. In this case, neat-SnO_2_ showed the highest responses to C_2_H_4_ and C_7_H_8_ than other sensors, consistent with the amount of surface acidity. The result indicated that surface acidic sites might play a more vital role in the combustion of C_2_H_4_ and C_7_H_8_ than surface oxygen ions [45].

**Figure 7:**
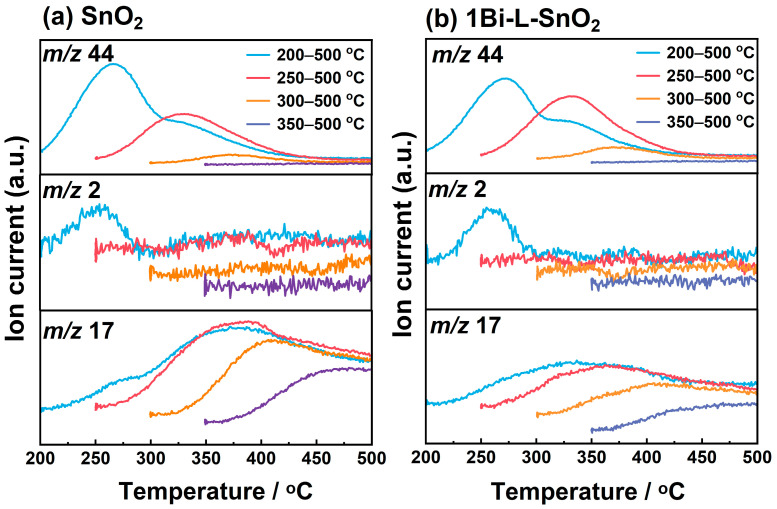
TPR spectra of (**a**) SnO_2_ and (**b**) 1Bi-L-SnO_2_ under O_2_/Ar flow after exposing 100 ppm ethanol/Ar during heating period from 200 °C, 250 °C, 300 °C, 350 °C, to 500 °C at mass numbers 44, 2, and 17.

The desorption signals on SnO_2_ and 1Bi-L-SnO_2_ surfaces after ethanol adsorption were investigated by ethanol-TPR measurements. Initially, ethanol molecules were respectively adsorbed on the materials at various temperatures (200 °C, 250 °C, 300 °C, and 350 °C) and then heated to 500 °C under Ar flow containing 21% of O_2_. The desorption signals at mass numbers 44 (the fragment of CH_3_CHO and CO_2_), 2 (H_2_), and 17 (H_2_O) were monitored during the heating period, as shown in Figure 7. Roughly, SnO_2_ and 1Bi-L-SnO_2_ materials showed similar TPR spectra. When ethanol was exposed at 200 °C, *m*/*z* 44 and *m*/*z* 2 exhibited distinct desorption peaks during 200–300 °C. According to the catalytic combustion result, no CO_2_ was produced at 200 °C and 250 °C, indicating the peak at *m*/*z* 44 was probably mainly caused by the desorption of CH_3_CHO. The peak at *m*/*z* 2 was assigned to the production of H_2_ arising from the deprotonation or dehydrogenation of ethanol. The phenomenon suggested that ethanol molecules could be dissociated to CH_3_CHO accompanied by the release of protons on SnO_2_ and 1Bi-L-SnO_2_ surfaces. When the materials adsorbed ethanol above 250 °C, *m*/*z* 44 and *m*/*z* 17 exhibited desorption peaks while no H_2_ was produced during the heating process, indicating ethanol molecules were further oxidized. As a result, ethanol molecules on SnO_2_ and 1Bi-L-SnO_2_ surfaces were mainly followed by dissociation and partial oxidation at 200 °C and 250 °C. Besides, the desorption amount at *m*/*z* 44 was drastically decreased when ethanol was exposed at 300 °C and 350 °C, illustrating ethanol molecules were easily oxidized and desorbed from the material surface at high temperatures.

### 3.5. Sensing Mechanism

Based on the above discussions, we proposed the possible sensing mechanism of SnO_2_ and Bi_2_O_3_-loaded SnO_2_ gas sensors. Firstly, the reaction pathway of ethanol combustion on the SnO_2_ surface was investigated as the representative of oxygenated VOCs. For neat-SnO_2_, at 200 °C and 250 °C, ethanol molecules were initially adsorbed and then partially dissociated to ethoxides or CH_3_CHO via deprotonation or dehydrogenation, respectively. Meanwhile, the dissociated protons could remain on the SnO_2_ surface by bonding with oxygen ions. Next, part of the ethoxides and CH_3_CHO molecules were further oxidized to CO_2_ and intermediates (CH_3_COOH, CO, etc.) by combustion reaction as the increase in temperature (300 °C and 350 °C). On the other hand, a small amount of ethanol molecules was converted to C_2_H_4_ and H_2_O by dehydration at the acidic sites of SnO_2_. In this case, the dissociation and partial oxidation of ethanol exhibited a significant effect on the sensitivities especially at 200 °C and 250 °C. According to the results of O_2_-TPD measurements, the surface lattice oxygen of SnO_2_ tended to be active at higher temperatures. Hence, the surface oxygen ions were major active species for ethanol adsorption.

**Figure 8:**
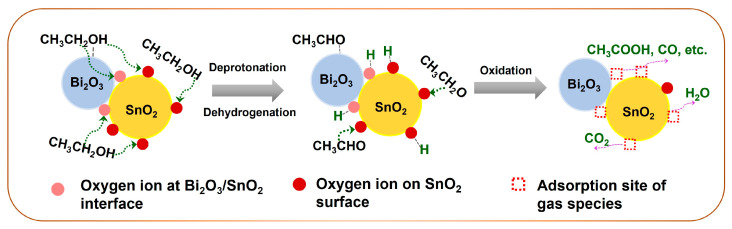
Schematic illustration of ethanol oxidation route on the 1Bi-L-SnO_2_ material surface.

Figure 8 shows the reaction pathway of ethanol combustion on the 1Bi-L-SnO_2_ surface. 1Bi-L-SnO_2_ sensor showed a larger amount of surface oxygen ions, as demonstrated in O_2_-TPD profiles. At 200 °C and 250 °C, the increased basic oxygen ions (mainly O^−^) could attract the adsorption of ethanol molecules by promoting the cleavage of acidic α-H and O-H bonding. Subsequently, the ethoxides and CH_3_CHO were partially oxidized with the increase in temperature, which was similar to neat-SnO_2_. Additionally, the catalytic combustion results indicated 1Bi-L-SnO_2_ showed a declined production of CO_2_ than neat-SnO_2_ material, revealing the improvement in surface oxygen ions improved the partial combustion of ethanol. In this context, the construction of Bi_2_O_3_/SnO_2_ interfaces mainly improved the initial adsorption, dissociation, and partial combustion of ethanol molecules. As a result, the 1Bi-L-SnO_2_ sensor showed higher sensitivity and faster response speed to ethanol than neat-SnO_2,_ particularly at 200 °C and 250 °C. Furthermore, oxygen vacancies would be exposed after consuming the surface oxygen ions, especially at high temperatures, accelerating the adsorption of oxygen and gas molecules. In addition, catalytic combustion and TPR measurements confirmed the active combustion and desorption of ethanol on material surfaces at 300–350 °C, leading to a smaller change in the electrical resistance than at 200–250 °C. Hence, the SnO_2_ and 1Bi-L-SnO_2_ sensors showed fast response speeds but weak sensitivities to ethanol at high temperatures. Moreover, the production of C_2_H_4_ almost disappeared due to the declined exposure of surface acidic sites by the Bi_2_O_3_-loading process. With the increasing concentration of Bi_2_O_3_ (3Bi-L-SnO_2_ sensor), the amount of surface oxygen ions was further increased, while the excessively dispersed Bi_2_O_3_ particles on the SnO_2_ surface disturbed the adsorption of gas molecules on SnO_2_, suppressing the further oxidation. Therefore, the 3Bi-L-SnO_2_ sensor showed a higher response speed but lower sensitivity to oxygenated VOCs than the 1Bi-L-SnO_2_ sensor.

As the major intermediate during ethanol combustion, the oxidation of CH_3_CHO might show similar productions with that of ethanol combustion, such as CH_3_COOH and CO. In addition, the reaction pathway of methanol combustion frequently consisted with ethanol due to the same functional group. For example, CH_3_OH molecules can be dissociated to HCHO by dehydrogenation and further oxidized to HCOOH, CO, and CO_2_. For acetone combustion, we could not observe the main intermediates by catalytic combustion measurement except a little CH_3_CHO at 300 °C and 350 °C. However, the acidic α-H bonding of acetone might be cleavage by the basic adsorption ions to form CH_3_COCH_2_^−^ and protons. Subsequently, CH_3_CHO and CH_3_COOH might be produced during acetone combustion like ethanol combustion [46,47]. In addition, the catalytic combustion results confirmed the combustion of acetone on the 1Bi-L-SnO_2_ surface exhibited a similar phenomenon to that of ethanol, which was mainly in accordance with the adsorption–desorption and partial combustion. As a result, 1Bi-L-SnO_2_ showed outstanding sensitivity and selectivity to oxygenated VOCs due to the improvement in surface oxygen ions.

## 4. Conclusions

In this experiment, Bi_2_O_3_-mixed SnO_2_ and Bi_2_O_3_-loaded SnO_2_ materials were synthesized for fabricating efficient gas sensors toward multiple oxygenated VOCs. Bi_2_O_3_ particles were uniformly dispersed on the SnO_2_ surface for Bi_2_O_3_-loaded SnO_2_ materials, leading to the construction of abundant Bi_2_O_3_/SnO_2_ interfaces. While agglomerated Bi_2_O_3_ particles were physically mixed with SnO_2_ NPs in Bi_2_O_3_-mixed SnO_2_ samples. O_2_-TPD spectra revealed the increased surface oxygen ions of Bi_2_O_3_-loaded SnO_2_ materials than that of SnO_2_ and Bi_2_O_3_-mixed SnO_2_ materials. 1Bi-L-SnO_2_ sensor showed improved sensitivity and selectivity to tested oxygenated VOCs, particularly at 200 °C and 250 °C. While the Bi_2_O_3_-mixed SnO_2_ sensor exhibited similar sensing properties to the SnO_2_ sensor. According to the results of catalytic combustion and TPR measurements, the detected sensitivities to ethanol of SnO_2_ and 1Bi-L-SnO_2_ sensors were primarily caused by the adsorption and partial oxidation of ethanol molecules. Meanwhile, the Bi_2_O_3_-loading process improved the partial combustion of ethanol on the SnO_2_ surface. The phenomenon indicated the effect of different distributions of Bi_2_O_3_ on the sensing performance of SnO_2_-based sensors. The abundant Bi_2_O_3_/SnO_2_ interfaces formed by the uniform dispersion of Bi_2_O_3_ particles were responsible for the enhancement of surface oxygen ions and sensing properties. The research provided a promising application for achieving sensitive and selective gas sensors for total oxygenated VOC detection by modulating the surface properties of sensing materials using foreign additives.

## Data Availability

The original contributions presented in the study are included in the article and Supplementary Materials. Further inquiries can be directed to the corresponding author.

## Supplementary Materials

The following supporting information can be downloaded at: https://www.mdpi.com/article/10.3390/nano14242032/s1, Figure S1: Explanation of operating processes during (a) O_2_-TPD-MS (b) NH_3_-TPD-MS. Figure S2. SEM-EDS mapping images of Sn and Bi elements for (a) 3Bi-M-SnO_2_ and (b) 3Bi-L-SnO_2_ samples. Figure S3. The electrical resistances in synthetic air of as-fabricated gas sensors at 200 °C, 250 °C, 300 °C, and 350 °C. Figure S4: Dynamic time-dependence response curves of gas sensors to 5 ppm of (a) CH_3_CH_2_OH, (b) CH_3_CHO, (c) CH_3_COCH_3_, (d) CH_3_OH at 200 °C. Figure S5: Dynamic time-dependence response curves of gas sensors to 5 ppm of (a) CH_3_CH_2_OH, (b) CH_3_CHO, (c) CH_3_COCH_3_, (d) CH_3_OH at 250 °C. Figure S6: Dynamic time-dependence response curves of gas sensors to 5 ppm of (a) CH_3_CH_2_OH, (b) CH_3_CHO, (c) CH_3_COCH_3_, (d) CH_3_OH at 300 °C. Figure S7: Dynamic time-dependence response curves of gas sensors to 5 ppm of (a) CH_3_CH_2_OH, (b) CH_3_CHO, (c) CH_3_COCH_3_, (d) CH_3_OH at 350 °C. Figure S8. The temperature-dependence of (a) CH_3_CH_2_OH consumption, (b) CO_2_, (c) CH_3_CHO, and (d) C_2_H_4_ yields during ethanol combustion on the surfaces of SnO_2_ and 1Bi-L-SnO_2_ particles from 200 °C to 350 °C. Figure S9. The temperature-dependence of (a) CH_3_COCH_3_ consumption, (b) CO_2_, and (c) CH_3_CHO yields during acetone combustion on the surfaces of SnO_2_ and 1Bi-L-SnO_2_ particles from 200 °C to 350 °C. Figure S10. (a) NH_3_-TPD profiles of as-prepared materials; NH_3_-TPD combined with mass spectrometer spectra of (b) SnO_2_, (c) 1Bi-M-SnO_2_, (d) 3Bi-M-SnO_2_, (e) 1Bi-L-SnO_2_ and (f) 3Bi-L-SnO_2_ materials. Table S1. Average crystallite size, BET, and BJH results of SnO_2_, 1Bi-M-SnO_2_, 3Bi-M-SnO_2_, 1Bi-L-SnO_2_, 3Bi-L-SnO_2_ materials. Table S2. Response time of SnO_2_ and Bi_2_O_3_-loaded sensors to 5 ppm of ethanol at 200–350 °C.

## References

[1] Wang W., Yan Y., Fang H., Li J., Zha S., Wu T. (2023). Volatile organic compound emissions from typical industries: Implications for the importance of oxygenated volatile organic compounds. Atmos. Pollut. Res..

[2] Gilman J.B., Lerner B.M., Kuster W.C., Goldan P.D., Warneke C., Veres P.R., Roberts J.M., Gouw J.A., Burling I.R., Yokelson R.J. (2015). Biomass burning emissions and potential air quality impacts of volatile organic compounds and other trace gases from fuels common in the US. Atmos. Chem. Phys..

[3] Mellouki A., Wallington T.J., Chen J. (2015). Atmospheric chemistry of oxygenated volatile organic compounds: Impacts on air quality and climate. Chem. Rev..

[4] Ran L., Zhao C.S., Xu W.Y., Lu X.Q., Han M., Lin W.L., Yan P., Xu X.B., Deng Z.Z., Ma N. (2011). VOC reactivity and its effect on ozone production during the HaChi summer campaign. Atmos. Chem. Phys..

[5] Wang F., Ho S.S.H., Man C.L., Qu L., Wang Z., Ning Z., Ho K.F. (2024). Characteristics and sources of oxygenated VOCs in Hong Kong: Implications for ozone formation. Sci. Total Environ..

[6] Matheus C.R.V., Aguiar E.F.S. (2020). The role of MPV reaction in the synthesis of propene from ethanol through the acetone route. Catal. Commun..

[7] Sun Y., Zhang X., Li N., Xing X., Yang H., Zhang F., Cheng J., Zhang Z., Hao Z. (2019). Surface properties enhanced Mn_x_AlO oxide catalysts derived from Mn_x_Al layered double hydroxides for acetone catalytic oxidation at low temperature. Appl. Catal. B-Environ. Energy.

[8] Lee J., Jung Y., Sung S.H., Lee G., Kim J., Seong J., Shim Y.S., Jun S.C., Jeon S. (2021). High-performance gas sensor array for indoor air quality monitoring: The role of Au nanoparticles on WO_3_, SnO_2_, and NiO-based gas sensors. J. Mater. Chem. A.

[9] Li C., Kim K., Fuchigami T., Asaka T., Kakimoto K.I., Masuda Y. (2023). Acetone gas sensor based on Nb_2_O_5_@SnO_2_ hybrid structure with high selectivity and ppt-level sensitivity. Sens. Actuators B Chem..

[10] Sui N., Zhang P., Zhou T.T., Zhang T. (2021). Selective ppb-level ozone gas sensor based on hierarchical branch-like In_2_O_3_ nanostructure. Sens. Actuators B Chem..

[11] Xu H., Gong Z.X., Huo L.Z., Guo C.F., Yang X.J., Wang Y.X., Luo X.P. (2023). Zinc Oxide-Loaded Cellulose-Based Carbon Gas Sensor for Selective Detection of Ammonia. Nanomaterials.

[12] Suematsu K., Harano W., Yamasaki S., Watanabe K., Shimanoe K. (2020). One-trillionth level toluene detection using a dual-designed semiconductor gas sensor: Material and sensor-driven designs. ACS Appl. Electron. Mater..

[13] Maekawa T., Tamaki J., Miura N., Yamazoe N., Matsushima S. (1992). Development of SnO_2_-based ethanol gas sensor. Sens. Actuators B Chem..

[14] Ren H., Zhao W., Wang L., Ryu S.O., Gu C. (2015). Preparation of porous flower-like SnO_2_ micro/nano structures and their enhanced gas sensing property. J. Alloys Compd..

[15] Zhang S., Pu Y., Cao S., Zhu D. (2023). SnO_2_ nanoparticles derived from metal–organic precursors as an acetaldehyde gas sensor with ppb-level detection limit. ACS Appl. Nano Mater..

[16] Suematsu K., Hiroyama Y., Watanabe K., Shimanoe K. (2022). Amplifying the receptor function on Ba_0.9_La_0.1_FeO_3_-SnO_2_ composite particle surface for high sensitivity toward ethanol gas sensing. Sens. Actuators B Chem..

[17] Zhu X., Cao P., Li P., Yu Y., Guo R., Li Y., Yang H. (2024). Bimetallic PtAu-Decorated SnO_2_ Nanospheres Exhibiting Enhanced Gas Sensitivity for Ppb-Level Acetone Detection. Nanomaterials.

[18] Zong S., Zhang Y., Cao J., Qin C., Bala H., Wang Y. (2024). Hydrothermal Synthesis of SnO_2_ with Different Morphologies as Sensing Materials for HCHO Detection. Langmuir.

[19] Jinkawa T., Sakai G., Tamaki J., Miura N., Yamazoe N. (2000). Relationship between ethanol gas sensitivity and surface catalytic property of tin oxide sensors modified with acidic or basic oxides. J. Mol. Catal. A-Chem..

[20] Torai S., Ueda T., Kamada K., Hyodo T., Shimizu Y. (2023). Effects of addition of Cu_x_O to porous SnO_2_ microspheres prepared by ultrasonic spray pyrolysis on sensing properties to volatile organic compounds. Chemosensors.

[21] Kumar A., Mukasyan A.S., Wolf E.E. (2011). Combustion synthesis of Ni, Fe and Cu multi-component catalysts for hydrogen production from ethanol reforming. Appl. Catal. A-Gen..

[22] Suematsu K., Ma N., Yuasa M., Kida T., Shimanoe K. (2015). Surface-modification of SnO_2_ nanoparticles by incorporation of Al for the detection of combustible gases in a humid atmosphere. RSC Adv..

[23] Suematsu K., Shin Y., Ma N., Oyama T., Sasaki M., Yuasa M., Kida T., Shimanoe K. (2015). Pulse-driven micro gas sensor fitted with clustered Pd/SnO_2_ nanoparticles. Anal. Chem..

[24] Zhang H., Guo S., Zheng W., Wang H., Li H.Y., Yu M.H., Chang Z., Bu X.H., Liu H. (2023). Facile engineering of metal–organic framework derived SnO_2_-ZnO composite based gas sensor toward superior acetone sensing performance. Chem. Eng. J..

[25] Dong Z.M., Xia Q., Ren H., Shang X., Lu X., Joo S.W., Huang J. (2023). Preparation of hollow SnO_2_/ZnO cubes for the high-performance detection of VOCs. Ceram. Int..

[26] Acharyya S., Bhowmick P.K., Guha P.K. (2023). Selective identification and quantification of VOCs using metal nanoparticles decorated SnO_2_ hollow-spheres based sensor array and machine learning. J. Alloys Compd..

[27] Niwa M., Igarashi J.Y. (1999). Role of the solid acidity on the MoO_3_ loaded on SnO_2_ in the methanol oxidation into formaldehyde. Catal. Today.

[28] Yan H., Liu T., Lv Y., Xu X., Xu J., Fang X., Wang X. (2024). Doping SnO_2_ with metal ions of varying valence states: Discerning the importance of active surface oxygen species vs. acid sites for C_3_H_8_ and CO oxidation. Phys. Chem. Chem. Phys..

[29] Lu B., Ma S., Liang S., Wang Z., Liu Y., Mao S., Ban H., Wang L., Wang Y. (2023). Efficient Conversion of Ethanol to 1-Butanol over Adjacent Acid–Base Dual Sites via Enhanced C–H Activation. ACS Catal..

[30] Chauhan A., Verma R., Dhatwalia J., Kumari A., Dutta V., Chandrasekaran G., Ghotekar S., Kaur M., Vignesh J., Thakur S. (2024). Phyto-mediated synthesis of pure phase α-Bi_2_O_3_ nanostructures using *Rubus ellipticus* plant extract: Photocatalytic activity and antimicrobial efficacy. Biomass Convers. Biorefinery.

[31] Dai W., Wang P., Long J., Xu Y., Zhang M., Yang L., Zou J., Luo X., Luo S. (2023). Constructing robust Bi active sites in situ on α-Bi_2_O_3_ for efficient and selective photoreduction of CO_2_ to CH_4_ via directional transfer of electrons. ACS Catal..

[32] Moumen A., Zappa D., Poli N., Comini E. (2021). Catalyst–Assisted vapor liquid solid growth of α-Bi_2_O_3_ nanowires for acetone and ethanol detection. Sens. Actuators B Chem..

[33] Jiang S., Wang L., Hao W., Li W., Xin H., Wang W., Wang T. (2015). Visible-light photocatalytic activity of S-doped α-Bi_2_O_3_. J. Phys. Chem. C.

[34] Park S., Kim S., Sun G.J., Lee C. (2015). Synthesis, structure, and ethanol gas sensing properties of In_2_O_3_ nanorods decorated with Bi_2_O_3_ nanoparticles. ACS Appl. Mater. Interfaces.

[35] Cheng L., Li Y., Sun G., Cao J., Wang Y. (2023). Modification of Bi_2_O_3_ on ZnO porous nanosheets-assembled architecture for ultrafast detection of TEA with high sensitivity. Sens. Actuators B Chem..

[36] Zhang M., Liu K., Zhang X., Wang B., Xu X., Du X., Yang C., Zhang K. (2022). Interfacial energy barrier tuning of hierarchical Bi_2_O_3_/WO_3_ heterojunctions for advanced triethylamine sensor. J. Adv. Ceram..

[37] Tanaka K.I., Ozaki A. (1967). Acid-base properties and catalytic activity of solid surfaces. J. Catal..

[38] Guo Y., Liu B., Duan Z., Yuan Z., Jiang Y., Tai H. (2023). Batch fabrication of H_2_S sensors based on evaporated Pd/WO_3_ film with ppb-level detection limit. Mater. Chem. Phys..

[39] Suematsu K., Watanabe K., Tou A., Sun Y., Shimanoe K. (2018). Ultraselective toluene-gas sensor: Nanosized gold loaded on zinc oxide nanoparticles. Anal. Chem..

[40] Gonçalves F., Medeiros P.R., Eon J.G., Appel L.G. (2000). Active sites for ethanol oxidation over SnO_2_-supported molybdenum oxides. Appl. Catal. A-Gen..

[41] Sun J., Wang Y. (2014). Recent advances in catalytic conversion of ethanol to chemicals. ACS Catal..

[42] Yamazoe N., Fuchigami J., Kishikawa M., Seiyama T. (1979). Interactions of tin oxide surface with O_2_, H_2_O and H_2_. Surf. Sci..

[43] Guo Y., Liang J., Liu Y., Liu Y., Xu X., Fang X., Zhong W., Wang X. (2019). Identifying surface active sites of SnO_2_: Roles of surface O_2_^−^, O_2_^2−^ anions and acidic species played for toluene deep oxidation. Ind. Eng. Chem. Res..

[44] Yan L., Liu Y., Zha K., Li H., Shi L., Zhang D. (2017). Deep insight into the structure–activity relationship of Nb modified SnO_2_–CeO_2_ catalysts for low-temperature selective catalytic reduction of NO by NH_3_. Catal. Sci. Technol..

[45] Liu Y., Guo Y., Liu Y., Xu X., Peng H., Fang X., Wang X. (2017). SnO_2_ nano-rods promoted by In, Cr and Al cations for toluene total oxidation: The impact of oxygen property and surface acidity on the catalytic activity. Appl. Surf. Sci..

[46] Meziane I., Fenard Y., Delort N., Herbinet O., Bourgalais J., Ramalingam A., Heufer K.A., Battin-Leclerc F. (2023). Experimental and modeling study of acetone combustion. Combust. Flame.

[47] Zaki M.I., Hasan M.A., Pasupulety L. (2001). Surface reactions of acetone on Al_2_O_3_, TiO_2_, ZrO_2_, and CeO_2_: IR spectroscopic assessment of impacts of the surface acid-base properties. Langmuir.

