# Supplementary material for: Oxygenated VOC Detection Using SnO2 Nanoparticles with Uniformly Dispersed Bi2O3
Source: Nanomaterials (Basel). 2024 Dec 18;14(24):2032. doi: 10.3390/nano14242032 (PMC11678746; doi:10.3390/nano14242032)
Supplement: Supplementary file 1 [file nanomaterials-14-02032-s001.zip › nanomaterials-3371463-supplementary.pdf]

Supporting Information

Oxygenated VOC Detection Using SnO_2 Nanoparticles with Uniformly Dispersed Bi_2O_3

Haoyue Yang[1], Koichi Suematsu[2*], Felipe Hiroshi Mashiba[1], Ken Watanabe[2], and Kengo Shimanoe[2*]

[1]*Interdisciplinary Graduate School of Engineering Sciences, Kyushu University, Kasuga, Fukuoka 816-8580, Japan*

[2] *Department of Advanced Materials Science and Engineering, Faculty of Engineering Sciences, Kyushu University, Kasuga, Fukuoka 816-8580, Japan*

O_2-TPD measurements

Firstly, 0.2 g of sample were pressed and then ground to maintain the sizes of powders between 250 and 710 μm. The obtained powders were put in reaction chamber and preheated at 550 °C for 2 h under synthetic air flow to refresh the surface. After cooled down to 100 °C, the samples were purged by helium (He) for 1.5 h. Finally, the sample was heated to 550 °C under He flow with a rate of 10 °C/min, as shown in Fig S1a. The desorbed signal from material surface during the heating period was detected by mass spectrometer. Herein, the mass spectrometer monitored the signal at mass number 32 was assigned to O_2.

NH_3-TPD measurements

The procedure of NH_3-TPD was broadly similar with O_2-TPD. Before measurement, the pressed powders (250~710 μm) were preheated at 550 °C for 2 h and cooled down to 100 °C under air flow followed by purged using He for 1 h. Subsequently, NH_3/He flow was introduced for 30 min and then exposure to He flow for 30 min until the baseline was stable. Finally, the sample was heated to 550 °C under He flow with a rate of 10 °C/min, as shown in Fig S1b. The desorbed signal from material surface during the heating period was detected and recorded by TCD detector. Meanwhile, the signals at mass numbers 16 (the fragment of NH_3 and H_2O), 17 (NH_3 and the fragment of H_2O), 18 (H_2O), 32 (O_2), 14 (N_2) and 28 (N_2) were monitored by mass spectrometer.

Catalytic combustion measurements

Before measurement, the quantity of emitted gases was calibrated by neat nitrogen, oxygen, and standard gases. Firstly, 0.01g samples were pressed and then ground to maintain the sizes of powders between 250 and 710 μm. Next, the obtained particles were put into the reaction chamber and preheated at 550 °C for 3 h under synthesis air flow. Subsequently, 100 ppm of ethanol/air or acetone/air was introduced when the reaction chamber was cooled to 350 °C, 300 °C, 250 °C and 200 °C with the rate of 5 °C/min. Finally, the consumption of ethanol or acetone and the productions (C_2H_4, CH_3CHO and CO_2) come from gas combustion at different temperatures were identified by GC analyzer.

TPR measurements

The desorption signals on SnO_2 and 1Bi-L-SnO_2 surfaces after ethanol adsorption were investigated by TPR measurement. Firstly, 0.03g pretreated samples (250~710 μm) were put into reaction chamber and preheated at 550 °C for 3 h under O_2/Ar flow and then cooled to 200 °C, 250 °C, 300 °C and 350 °C with the rate of 5 °C/min, respectively. Subsequently, 100 ppm of ethanol/Ar was introduced for 1.5 h with the flow rate of 100 $cm^3 \cdot min^{-1}$ to adsorb ethanol molecules. Then the atmosphere was recovered to Ar flow containing 21% of O_2 for 6 h. Finally, the powders were heated to 500 °C with the rate of 5 °C/min under O_2/Ar flow. The emission of gases during the heating period on material surfaces were analyzed by QMS detector at mass numbers 2 (H_2), 17 (H_2O), 44 (the fragment of CH_3CHO and CO_2).

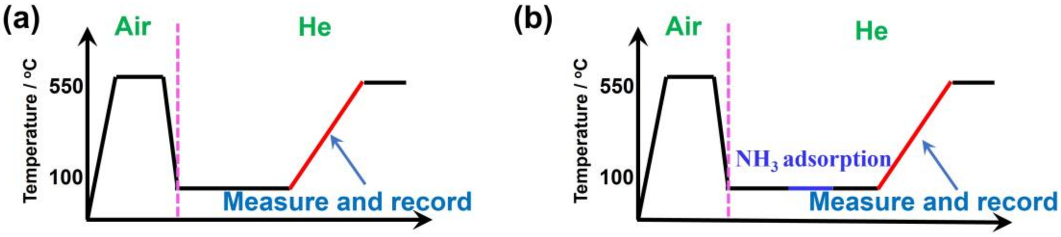

Figure S1. Explanation of operating processes during (a) O_2-TPD and (b) NH_3-TPD.

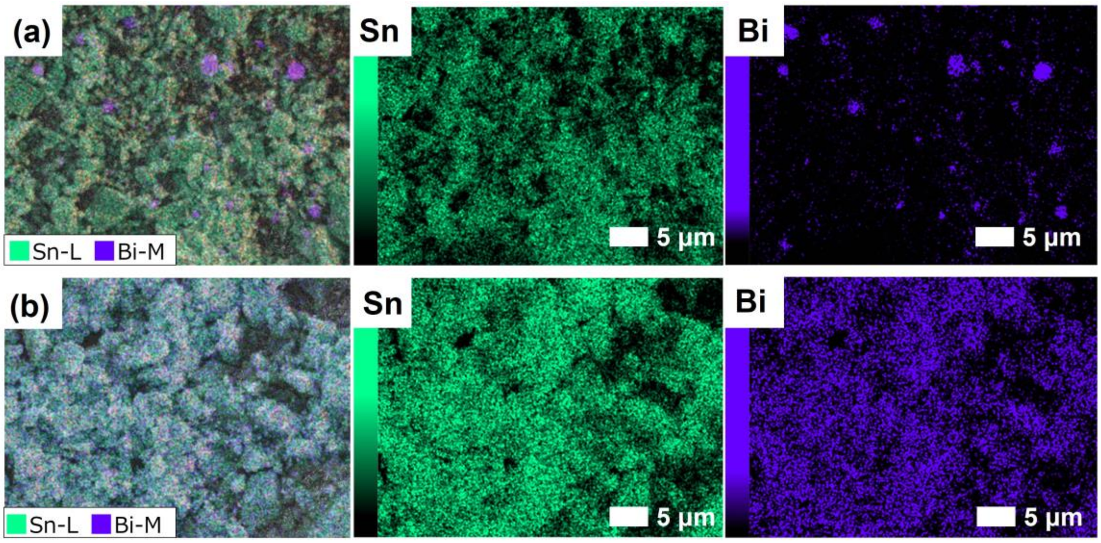

Figure S2. SEM-EDS mapping images of Sn and Bi elements for (a) 3Bi-M-SnO_2 and (b) 3Bi-L-SnO_2 samples.

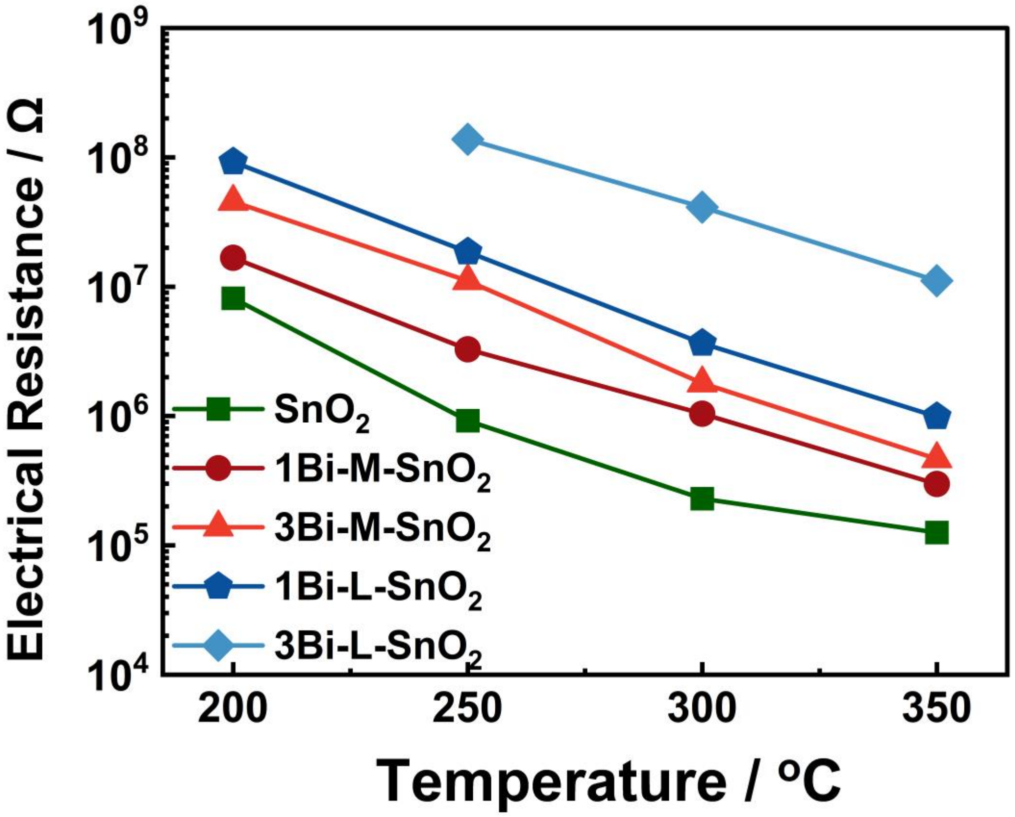

Figure S3. The electrical resistances in synthetic air of as-fabricated gas sensors at 200 ℃, 250 ℃, 300 ℃ and 350 ℃.

Obviously, the R_a of all sensors was reduced with the increasing operating temperature, consistent with the semiconductor behavior. Moreover, both Bi_2O_3-mixing and Bi_2O_3-loading processes increased the R_a values of SnO_2, and the R_a was enhanced with the rising amount of Bi_2O_3 particles. Meanwhile, Bi_2O_3-loaded SnO_2 exhibited larger R_a than that of Bi_2O_3-mixed SnO_2 sensors, which could be associated the generation of potential barrier at Bi_2O_3/SnO_2 interfaces.

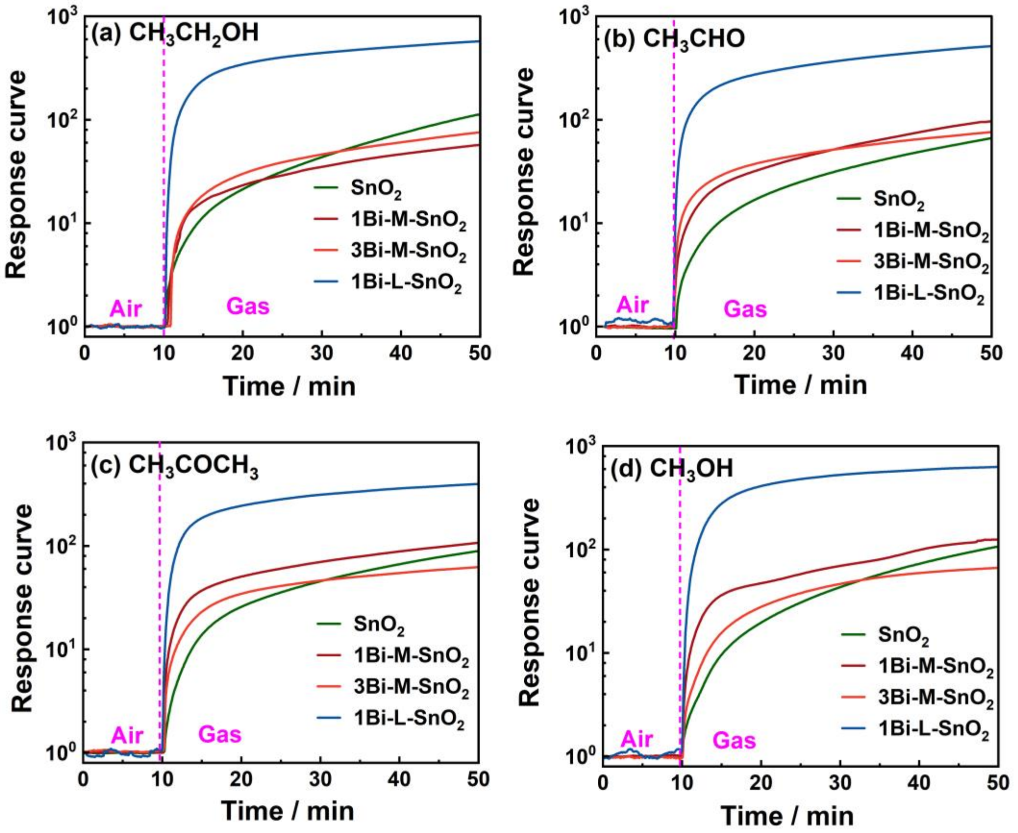

Figure S4. Dynamic time-dependence response curves of gas sensors to 5 ppm of (a) CH_3CH_2OH, (b) CH_3CHO, (c) CH_3COCH_3, (d) CH_3OH at 200 ℃.

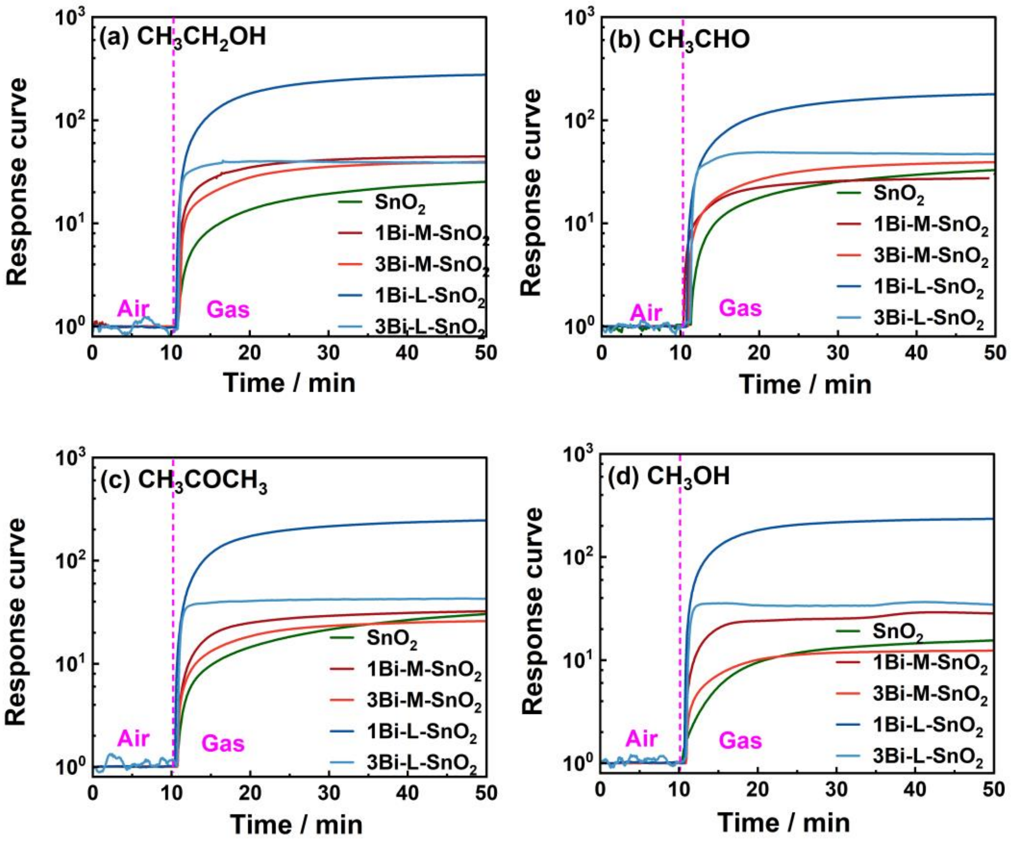

Figure S5. Dynamic time-dependence response curves of gas sensors to 5 ppm of (a) CH_3CH_2OH, (b) CH_3CHO, (c) CH_3COCH_3, (d) CH_3OH at 250 ℃.

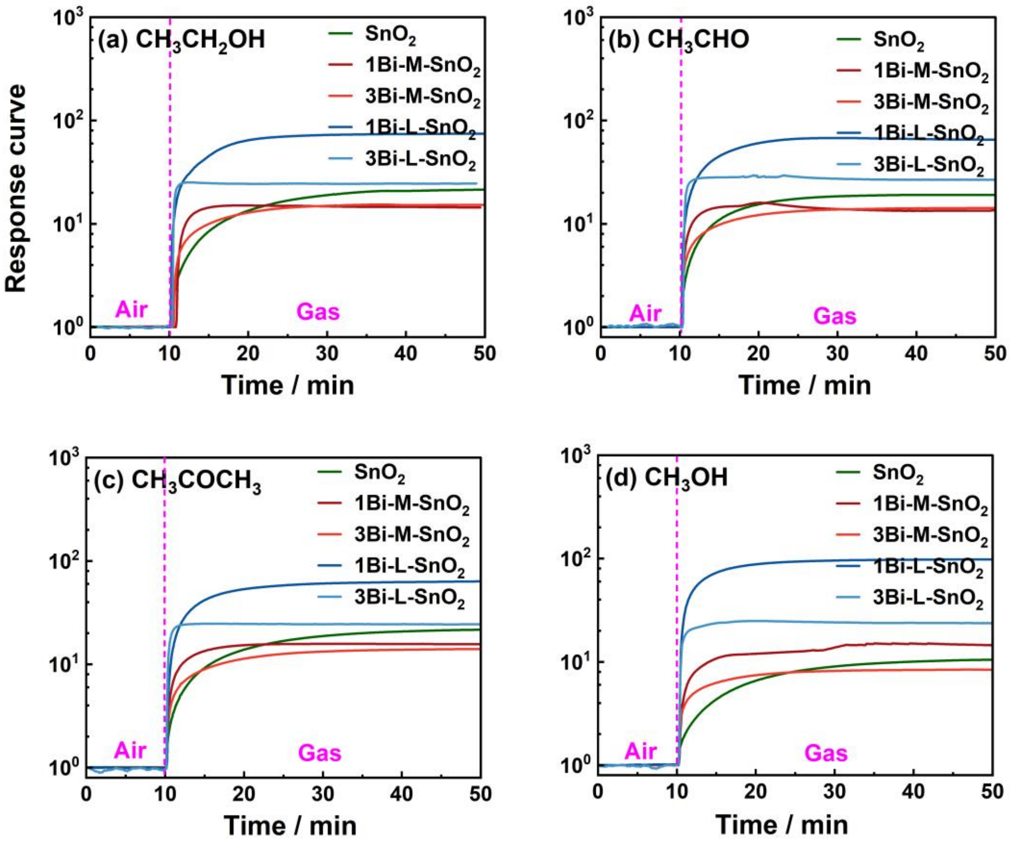

Figure S6. Dynamic time-dependence response curves of gas sensors to 5 ppm of (a) CH_3CH_2OH, (b) CH_3CHO, (c) CH_3COCH_3, (d) CH_3OH at 300 ℃.

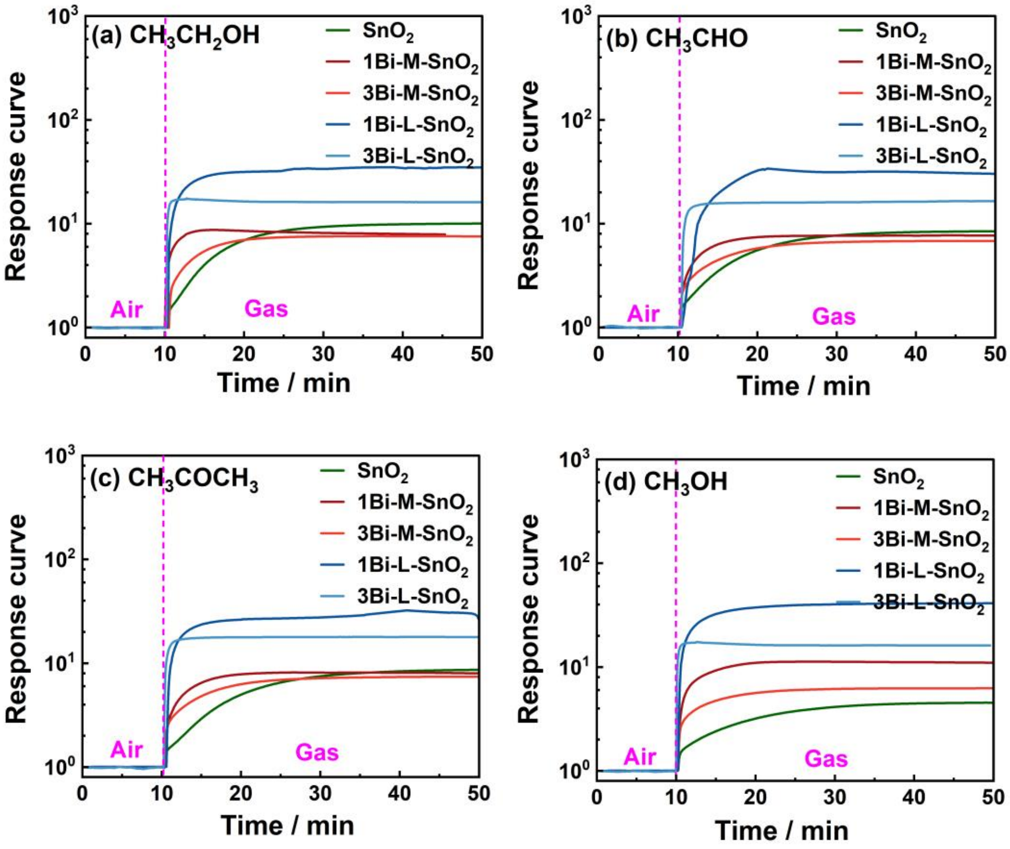

Figure S7. Dynamic time-dependence response curves of gas sensors to 5 ppm of (a) CH_3CH_2OH, (b) CH_3CHO, (c) CH_3COCH_3, (d) CH_3OH at 350 ℃.

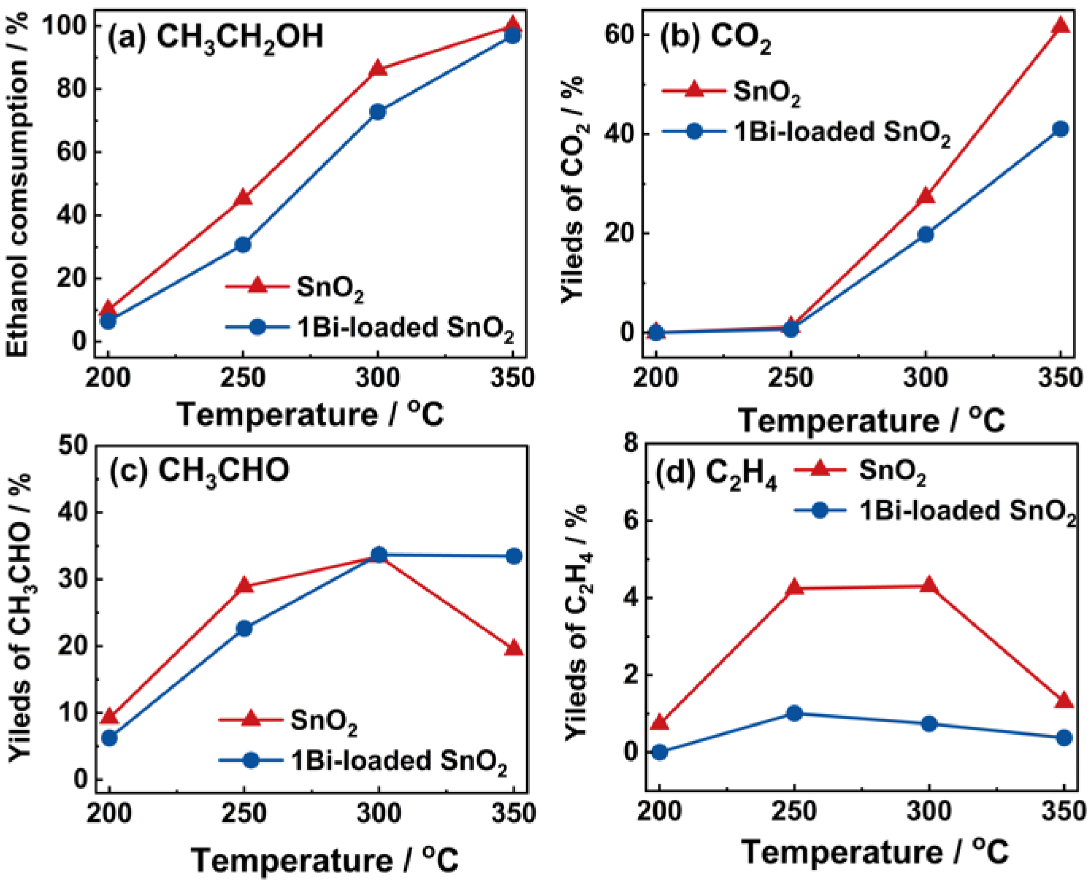

Figure S8. The temperature-dependance of (a) CH_3CH_2OH consumption, (b) CO_2, (c) CH_3CHO and (d) C_2H_4 yields during ethanol combustion on the surfaces of SnO_2 and 1Bi-L-SnO_2 particles from 200 °C to 350 °C.

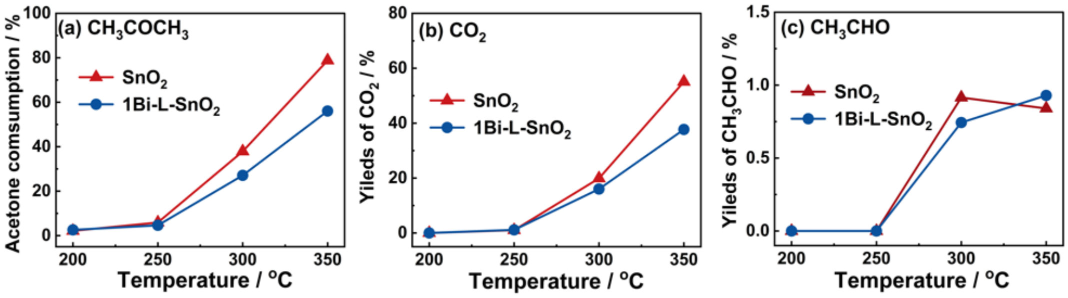

Figure S9. The temperature-dependance of (a) CH_3COCH_3 consumption, (b) CO_2 and (c) CH_3CHO yields during acetone combustion on the surfaces of SnO_2 and 1Bi-L-SnO_2 particles from 200 °C to 350 °C.

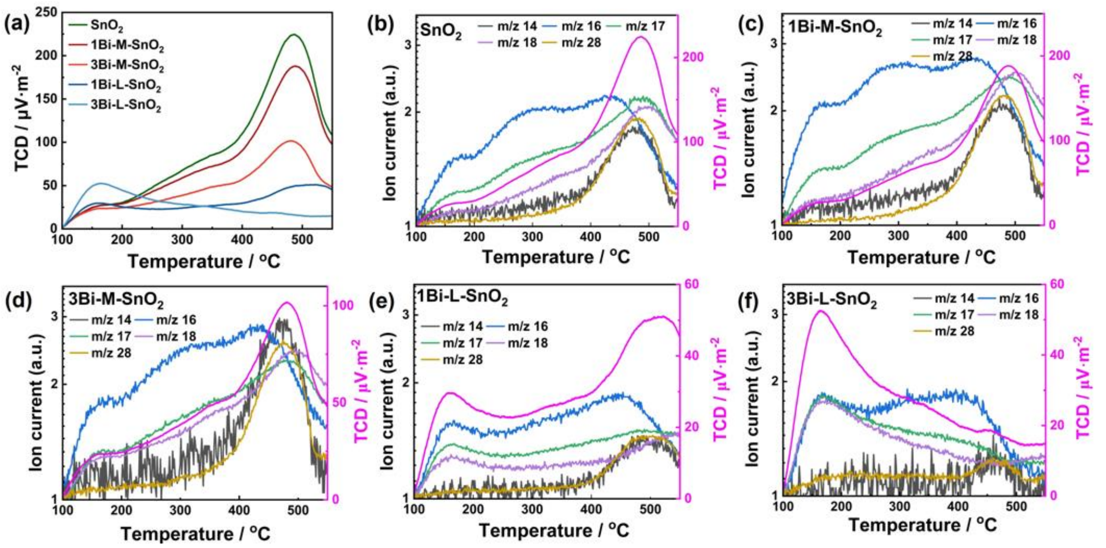

Figure S10. (a) NH_3-TPD profiles of as-prepared materials; NH_3-TPD combined with mass spectrometer spectra of (b) SnO_2, (c) 1Bi-M-SnO_2, (d) 3Bi-M-SnO_2, (e) 1Bi-L-SnO_2 and (f) 3Bi-L-SnO_2 materials.

Figure S10a showed the NH_3-TPD profiles of obtained materials. The desorption amounts recorded by TCD detector were divided their specific surface area. The peaks of NH_3 on the surfaces of as-prepared materials could be divided to 100–200 °C and 200–550 °C. Obviously, the desorption amount of NH_3 on SnO_2 surface was gradually declined with the addition of Bi_2O_3 particles. Interestingly, 3Bi-L-SnO_2 material showed slightly larger desorption amounts at 100–200 °C and extremely low desorption at higher temperature. The emitted gases from materials surfaces were further analyzed by mass spectrometer, as depicted in Figure S10b-f. At 100–400 °C, both m/z 16 and m/z 17 showed larger desorption peaks than m/z 18, proving the emitted gases were mainly assigned to the desorption of NH_3 molecules combined with a small amount of H_2O. Additionally, the distinct peaks at m/z 14 and m/z 28 during 400–550 °C were assigned to the generation of N_2 caused by the decomposition of NH_3. And the comparable intensity between m/z 17 and m/z 18 suggested the production of H_2O. Therefore, the desorption peak at 100–200 °C was mainly ascribed to the desorption of molecular NH_3 and H_2O, and the peak above 200 °C could be assigned to the physical and chemical adsorption of NH_3 on the acid sites of SnO_2. In this context, the decreased desorption amounts during 200–550 °C on Bi_2O_3-mixed SnO_2 and Bi_2O_3-loaded SnO_2

materials indicated the reduced acidic sites of SnO_2 for NH_3 adsorption. Moreover, 3Bi-L-SnO_2 material showed distinct peak at m/z 18 during 100–200 °C, indicating the improved adsorption of molecular H_2O. Consequently, the exposure of acid sites on the surface of SnO_2 was declined by the introduction of Bi_2O_3 particles.

Table S1. Average crystallite size, BET and BJH results of SnO_2, 1Bi-M-SnO_2, 3Bi-M-SnO_2, 1Bi-L-SnO_2, 3Bi-L-SnO_2 materials.

Sample	Average crystallite size (nm) [a]	S_{BET} (m^2g^{-1}) [b]	V_{BJH} (cm^3g^{-1})$\times10^{-2}$ [c]
SnO_2	12.03	13.7	4.16
1Bi-M-SnO_2	10.93	15.7	4.51
3Bi-M-SnO_2	13.23	17.5	4.89
1Bi-L-SnO_2	10.95	17.7	4.52
3Bi-L-SnO_2	11.25	17.8	4.78

[a] Average crystallite size: average crystallite size of SnO_2 calculated by Scherrer formula.

[b] S_{BET}: specific surface area calculated by BET method.

[c] V_{BJH}: pore volume calculated by BJH method.

Table S2. Response time of SnO_2 and Bi_2O_3-loaded sensors to 5 ppm of ethanol at 200–350 °C.

Sensor	200 °C	250 °C	300 °C	350 °C
SnO_2	37.1 min	29.1 min	20.9 min	16.3 min
1Bi-L-SnO_2	30.9 min	22.7 min	13.3 min	6.6 min
3Bi-L-SnO_2	--	3.7 min	1.8 min	0.4 min
